# COVID-19 impacts on healthcare access in sub-Saharan Africa: an overview

**DOI:** 10.1590/1678-9199-JVATITD-2023-0002

**Published:** 2023-07-03

**Authors:** Jean-Philippe Chippaux

**Affiliations:** 1 Paris Cité University, Research Institute for Development, Mother and child in tropical environment: pathogens, health system and epidemiological transition, Paris, France.

**Keywords:** COVID-19, pandemic, impact, health service, healthcare access, Sub-Saharan Africa

## Abstract

This overview aimed to describe the situation of healthcare access in sub-Saharan Africa, excluding South Africa, during the COVID-19 pandemic. A PubMed^®^ search from March 31, 2020, to August 15, 2022, selected 116 articles. Healthcare access and consequences of COVID-19 were assessed based on comparisons with months before its onset or an identical season in previous years. A general reduction of healthcare delivery, associated with the decline of care quality, and closure of many specialty services were reported. The impact was heterogeneous in space and time, with an increase in urban areas at the beginning of the pandemic (March-June 2020). The return to normalcy was gradual from the 3^rd^ quarter of 2020 until the end of 2021. The impact of COVID-19 on the health system and its use was attributed to (a) conjunctural factors resulting from government actions to mitigate the spread of the epidemic (containment, transportation restrictions, closures of businesses, and places of entertainment or worship); (b) structural factors related to the disruption of public and private facilities and institutions, in particular, the health system; and (c) individual factors linked to the increase in costs, impoverishment of the population, and fear of contamination or stigmatization, which discouraged patients from going to health centers. They have caused considerable socio-economic damage. Several studies emphasized some adaptability of the healthcare offer and resilience of the healthcare system, despite its unpreparedness, which explained a return to normal activities as early as 2022 while the COVID-19 epidemic persisted. There appears to be a strong disproportion between the moderate incidence and severity of COVID-19 in sub-Saharan Africa, and the dramatic impact on healthcare access. Several articles make recommendations for lowering the socioeconomic consequences of future epidemics to ensure better management of health issues.

## Background

The numbers of COVID-19 cases and deaths in sub-Saharan Africa (SSA), excluding South Africa which accounts for nearly half of the reported COVID-19 cases in SSA, have been lower than in other continents for reasons that remain unclear [1-6]. However, excess mortality (i.e., the number of deaths exceeding expected all-cause mortality) was particularly high - more than 100 times the mortality directly attributed to COVID-19 - suggesting a very strong deleterious synergy between morbidity factors [6, 7].

**Figure 1.**
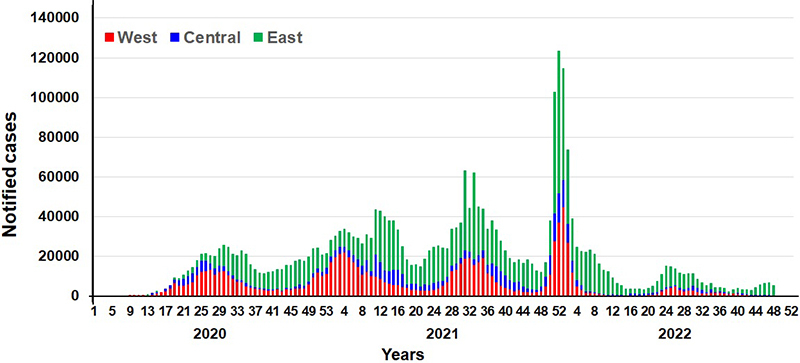
Weekly incidence of COVID-19 in the different regions of sub-Saharan Africa (except South Africa).

**Figure 2.**
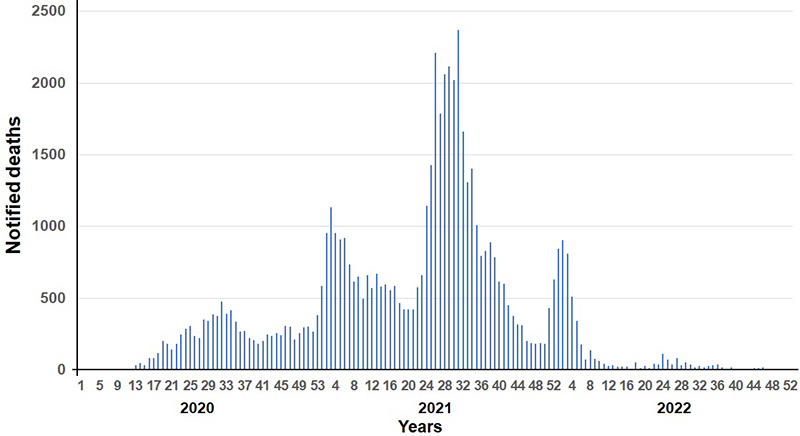
Weekly mortality of COVID-19 in the different regions of sub-Saharan Africa (except South Africa).

According to the COVID-19 dashboard edited and updated daily by the WHO Africa Regional Office, the first cases of COVID-19 were detected in Nigeria on February 28, 2020, and in Senegal on March 2, 2020, in travelers [8]. At least five epidemic waves occurred between March 2020 and August 2022, showing increasing incidence up to and including the fourth wave (November 2021 to March 2022), while mortality was highest during the second and third waves, from December 2020 to April 2021 (beta and then gamma variants), and June 2021 to September 2021 (delta variant), respectively (Figures 1 and 2). The first wave from March to June 2020, apparently mild, was accompanied by highly restrictive protective measures in most SSA countries [1], justified by international pressure and particularly pessimistic models of the impact of the pandemic on other diseases [9-15].

Access to healthcare requires that five specific dimensions be met: (a) availability, which reflects the adequacy between supply and demand, (b) accessibility, which implies geographical and logistical proximity between the patient and the health center, (c) accommodation, i.e. good quality services adapted to the needs of the public, (d) affordability for the population concerned, and (e) acceptability of the healthcare offered by people and health personnel [16].

A review of the literature during the COVID-19 pandemic period provided an overview of the situation of healthcare access and use of the healthcare system by the population in SSA countries excluding South Africa. This study aimed to identify the main impacts of reduced access to care by mentioning the causes identified by practitioners and researchers involved in the pandemic.

**Figure 3.**
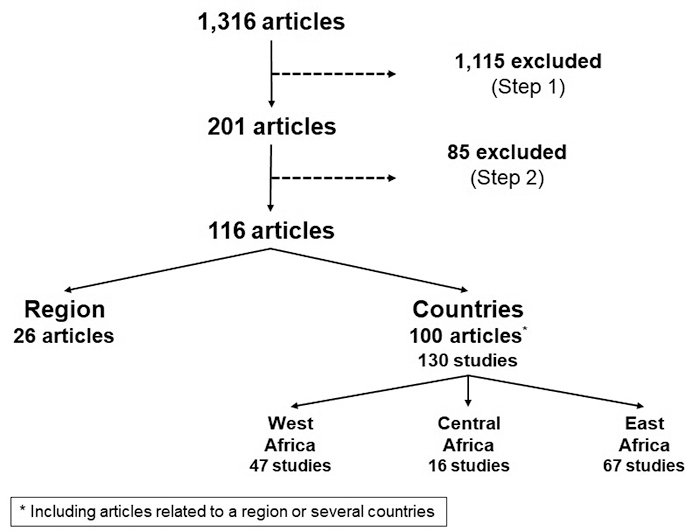
Flow chart of selected articles used in the review.

PubMed^®^ was searched using the keywords "COVID, Africa, Public health, Impact" targeting publications published from March 31, 2020, to August 15, 2022. Articles were selected in two steps, without language restriction (Figure 3). In step one, the title, abstract, and keywords were used to determine studies on health service utilization and access to healthcare, excluding South Africa. Studies from South Africa were not included because of its more developed health system and significantly higher resources than other countries in the region. In step two, a review of the objectives and methodology of each preselected article led to the rejection of editorials, comments on other articles, and responses to comments, as well as studies with incomplete data (more than 20% missing data) or small sample sizes (less than 30 individuals) considered insufficiently representative. The analysis of the articles selected in step two focused on healthcare system access, healthcare utilization, and the specific constraints of some vulnerable groups (people living with HIV (PLHIV), tuberculosis (TB) patients, children, women of childbearing age, sex workers, etc.). It was assessed based on comparisons with the months prior to the onset of COVID-19 or an identical season in previous years. The indicators used were the number of vaccinations, visits to various medical services (including prenatal visits and hospital deliveries), hospitalizations, and screening and management of endemic or chronic diseases (malaria, tuberculosis, HIV, diabetes, hypertension, cancers, etc.).

After the various stages of the selection process, 116 articles were retained out of the 1,316 identified at the first step (Figure 3). In addition to the 26 articles involving all or part of the sub-Saharan region, 100 articles described 130 studies conducted in 27 countries, more than half of which were in Ethiopia (16 studies), Ghana (11 studies), Kenya (14 studies), Nigeria (19 studies), and Uganda (11 studies) (Table 1). The central region of SSA was the least represented with 12% of the articles, *versus* 36% for West Africa and 52% for East Africa.

**Table 1.** List of SSA countries where studies regarding the impact of COVID-19 were carried out (111 publications selected on August 15, 2022).

Countries	August 15, 2022	References
Region	26	[1-3, 8, 10-13, 17-34]
Botswana	1	[19]
Burkina Faso	4	[19, 32-34]
Cameroon	4	[19, 35-37]
Congo	1	[19]
Cote d'Ivoire	2	[34,38]
DR Congo	3	[33, 39, 40]
Ethiopia	16	[15, 19, 32, 41-54]
Ghana	11	[19, 55-64]
Guinea	1	[65]
Guinea Bissau	1	[34]
Kenya	14	[19, 30, 33, 56, 66-76]
Malawi	4	[30, 75, 77, 78]
Mali	2	[30, 79]
Mozambique	5	[19, 80- 83]
Namibia	2	[19, 84]
Niger	1	[85]
Nigeria	19	[15, 19, 32, 33, 70, 74, 86- 98]
Uganda	11	[9, 30, 50, 76, 99-105]
Rwanda	3	[19, 106, 107]
Senegal	1	[108]
Sierra Leone	5	[34, 50, 109-111]
Somalia	1	[6]
Soudan	2	[19, 75]
Tanzania	3	[15, 19, 50]
Zambia	6	[4, 15, 19, 112- 114]
Zimbabwe	7	[19, 77, 79, 115- 119]

### Impact of COVID-19 on healthcare access

During the COVID-19 epidemic, healthcare services suffered a dramatic decline. The reduction in patient flow, sometimes up to 70%, affected all medical and surgical units - with significant cancellations or postponements of urgent responses - as well as preventive medicine units affecting the entire population [17-19, 32, 35-37, 41, 57, 66-68, 80, 85-87, 99, 109], and delays in the supply of drugs [19]. The number of consultations and hospitalizations decreased mainly in the second quarter of 2020 and then gradually increased again [35, 42]. Hospitalizations seem to have been more affected than consultations [17, 42, 46], as well as urban *versus* rural health facilities [17].

Paradoxically, despite the pessimistic predictions of models anticipating a doubling of malaria deaths in case of service interruption, access to basic clinical services in charge of malaria diagnosis was little disrupted in most countries with moderate or high malaria burden in 2020 thanks to the implementation of an adapted response [13]. For example, in Ghana, the number of malaria consultations among children and pregnant women decreased in March and April 2020 but straightened after that [57]. In Uganda, there was no difference between the observed and expected number of consultations for confirmed or suspected malaria cases [100].

Many TB screening appointments were missed due to reduced attendance at health facilities, leading to decrease diagnosis and initiation of treatment [43, 44, 88, 112]. In Zambia, specific intervention measures have resulted in the resumption of consultations in July 2020 and normalization in September 2021 [112].

Many PLHIV have refrained from counseling regardless of the reason and have suspended antiretroviral therapy (ART) [69, 101-103]. However, in Uganda, the number of people receiving prevention of vertical transmission of HIV has rapidly stabilized [101].

Across the SSA, there has been a decline in access to sexual and reproductive health services, institutional delivery, antenatal care, family planning, and HIV care. An increase in teenage pregnancies has been reported [20]. In Mozambique, overall antenatal visits decreased as well as cesarean sections and hospital deliveries, while home deliveries increased [80]. In contrast, in Kinshasa (Democratic Republic of Congo) maternal health services and vaccinations were poorly affected [39].

Childhood vaccinations (BCG, diphtheria-tetanus-pertussis, measles-rubella) have fallen sharply, leading to a worrying reduction in immunization coverage, raising fears of a resurgence of these diseases [36, 38, 45, 58, 80, 89, 108]. After the first wave, the trend reversed and returned to pre-March 2020 levels [68, 89].

In specialty services, including cardiology, urology, rheumatology, radiology, oncology, ophthalmology, and dentistry, visits have declined by more than 50% or even stopped in some places [21, 39, 59, 60, 65, 90-92, 116, 117].

People with chronic diseases had great difficulty accessing essential medicines during the COVID-19 lockdown, leading to deteriorating health conditions for most of them [69, 84, 94, 113].

The decline in blood donation began as soon as COVID-19 was announced, leading first to an interruption in mobile blood drive campaigns and then to the closure of blood donation centers in most countries [97]. In Nigeria, for example, the number of blood transfusions was dramatically reduced, partially offset by family replacement donations (FRDs) - the practice of drawing blood from a patient's family member. These decreases have been more pronounced for voluntary blood donations than for FDR, probably due to stronger incentives than pandemic-related constraints. However, it is not excluded that the family used a paid donor presented as an FDR [97].

The impact of COVID-19 on health services was very heterogeneous across localities - generally higher in urban than in rural areas - and over time, with a strong increase in the first months of the pandemic, and then according to the epidemic waves, which were not synchronous in different countries [3, 22, 35, 39, 42, 68, 89, 101, 112]. A comparative study using the same indicators and the same methodology showed the strong influence of the local context even if it is not always possible to determine the causes [77].

Vulnerable populations, including transgender people, sex workers, and drug users, were turned away from medical services, especially sexual and reproductive health services [81, 93].

However, several studies noted a gradual improvement between epidemic waves and a certain capacity to adapt the healthcare supply [39, 46, 57, 68, 82, 89, 100, 101, 112], and even a good resilience of the health system or the patients themselves [23, 47, 103, 105, 110, 113].

Access to healthcare was directly affected by the governmental measures taken to contain COVID-19 [24, 37, 38, 44, 48, 57, 69, 70, 84, 94, 99, 106, 108]. The implementation of physical distancing measures (containment, curfews, closure of businesses and places of entertainment or worship) impeded the movement of people and goods, and thus the seeking, provisioning, and delivery of healthcare, increasing tensions between healthcare personnel and patients [44, 57, 67, 69, 71, 99, 108, 116].

Inadequate health system preparedness was reported. It was due to (A) low availability of the health service, (B) inadequate resources and equipment, and (C) lack of appropriate testing, and therapeutic response for COVID-19 [26]. The reduction in staffing and the detour of human resources to meet the healthcare needs due to COVID-19 have led to a disorganization of the healthcare offer, in particular a prioritization of basic services with a general reduction in services or even suspension of care considered non-urgent (dentistry, physiotherapy, physical rehabilitation, etc.) [19, 57, 67, 99, 109]. In addition, there was a lack of personal protective equipment (disinfectant, masks, gloves, gowns, glasses, etc.) [57, 69]. This inability to meet the needs of the population has greatly contributed to their mistrust of institutions [25, 57].

A large proportion of the population refused to visit health centers for fear of contamination and stigma [19, 24, 25, 37, 38, 44, 48, 58, 64, 67, 70, 94-96, 99, 107, 109, 118, 119].

Many healthcare workers refused to care for patients for fear of contracting the virus themselves [37, 57, 50, 67, 78, 94, 99]. In addition, the increased workload and stigma they faced caused fatigue and stress, even depression, and increased absenteeism [78].

The exacerbation of pre-existing barriers (poverty, additional costs, and poor respect for confidentiality) has been highlighted by vulnerable populations to explain their reluctance to visit health centers [93, 118].

Finally, all the specific dimensions necessary to maintain healthcare access - as described by Penchansky and Thomas [16] were hampered by the pandemic (Table 2).

**Table 2.** Impact of COVID-19 on each of the components of healthcare access (definitions of healthcare access according to Penchansky & Thomas, 1981 [16]).

Dimension	Definition	Obstruction due to COVID-19	References
Availability	Adequacy between the supply and the demand	Closure of some health centers Organization worsening Health personnel overwork Shortage of health products Delay/cancellation of health interventions	[24, 28, 55, 61, 64, 67, 69, 74, 95, 102]
Accessibility	Relationship between the location of service or supply and the location of clients	Transportation restrictions and cost Confinement	[24, 44, 48, 55, 61, 64, 70, 71, 84, 94, 99, 106-108, 116]
Accommodation	Relationship between the service or supply resources, and the client’s ability and perception of their appropriateness	Complexity of COVID-19 protocols Disbelief from patients Reallocation of resources Bureaucracy	[26, 48, 61, 63, 64, 84, 93, 118, 119]
Affordability	Relationship between cost of services and client's income	Cost increase Loss of purchasing power	[33, 37, 44, 55, 70, 94, 96, 99]
Acceptability	Relationship between the client’s attitude and reaction and provider’s practice	Fear of contamination by COVID-19 Fear of being diagnosed positive Fear of stigma Loss of trust in health personnel Distrust of Western medicine Stress or depression Infodemic	[12, 19, 24, 25, 34, 37, 44, 48, 52, 55, 58, 61, 64, 67, 70, 74, 94, 95,107, 118, 119]

Nearly all studies showed a sharp decline in healthcare activity and access during the early months of the pandemic, followed by a gradual return to normal starting in the third quarter of 2020 and continuing through the end of 2021 (Table 3). The heterogeneity of the impact was significant, both temporally - based on epidemic waves that did not occur simultaneously across SSA - and spatially, according to different environmental and socioeconomic contexts. Although inadequate reporting and low frequency of reverse transcriptase-polymerase chain reaction (RT-PCR) testing of COVID-19 samples did not provide an accurate picture of the incidence and severity of the epidemic in SSA, no correlation appeared to exist with its impact on the healthcare system and its utilization. The causes remain speculative: differences in the socioeconomic environment, variable performance of the efficacy reporting system or the diagnosis and screening policy, and restrictive prevention measures [2, 3, 5]. However, it is possible to highlight that the incidence and mortality of the first wave, between March and June 2020, were barely noticeable, whereas they increased in the following waves (Figures 1 and 2), while healthcare access, dramatic at the beginning of the epidemic, tended to normalize. As soon as the first cases of COVID-19 appeared, drastic protective measures were taken in most countries: border closure, containment or curfew, travel and transportation bans, and business shutting down [1]. However, these measures were gradually reduced until they disappeared. It is questionable whether the governmental measures were successful in containing the epidemic in its early stages, or in delaying its expansion, whereas the gradual mitigation of the confinements from the second quarter of 2020 would have led to an increase in cases [1, 2, 5, 12].

**Table 3.** Decrease in access to health services in sub-Saharan Africa between April 2020 and June 2022 (expressed as % of activities excluding pandemic or wave of transmission).

Countries	OMS-AFRO	Cameroon	Cote d'Ivoire	Ethiopia	Ghana	Guinea	Kenya	Malawi	Mozambique	Niger	Nigeria	Uganda	Rwanda	Senegal	Sierra Leone	Zambia	Zimbabwe	References
Services																		
Immunization		-40- -60	-26- -99	+15- -12	-11- -47				-20	-61	-20	-9- -16		-42- -50				[17, 36, 38, 45, 50, 63, 80, 85, 89, 101, 108]
Outpatients				+1- -57		-71	-25					-50						[46, 65, 67, 103]
Pediatric outpatients		-34- -52																[27, 36]
Inpatients				-10- -73		-75	-14								-15			[46, 65, 67, 109]
Pediatric inpatients	-17	-25									-50	-14			-9			[17, 35, 37, 77, 87, 99, 109]
Family planning				-98- +47	-50				-28									[20, 41, 46, 63, 80, 93]
Prenatal visits	-2- -6	-34- -45		-7					-26			-60						[20, 27, 35, 36, 48, 80, 101]
Stillbirth rate				+19								+36					-7	[54, 99, 115]
Ophthalmology											-46							[92]
Emergencies				+19- -47														[46, 54]
Accidents				-36							-41							[54, 98]
Dialysis				-53														[46]
Deliveries	-2- -6				-35						-50	-5					-14	[17, 20, 63, 86, 101, 115]
Planned surgery				-92- -70														[41, 116]
Diabetes / HTA consultations																		[39, 49]
Malaria diagnosis									-3- -7			-9	-5					[28, 57, 100, 107]
HIV consultations and/or decrease ART	-43			-22								-34				-36		[20, 28, 29, 54, 55, 79, 93, 102, 103, 112]
Rabies consultations			-38- -45															[38]
TB diagnosis				-11					-15		-73				-25	-22		[28, 43, 82, 88, 93, 110, 112]
TB management				-17- -70							-72							[42-44, 88, 93]
Cancer diagnosis				-51- -99														[54]

In any case, the authors are unanimous in describing the socioeconomic disorganization caused by the government measures, making them unacceptable to the population to the point that they were quickly relaxed. Three types of factors explained the decline in healthcare access: conjunctural, linked to government measures for the mitigation of transmission; structural, resulting from the dysfunction of the health system; and individual, involving the reactions of health personnel and the population.

### Conjunctural factors

The Public Health and Social Measures Severity (PHSM) index can be applied to the governmental response to the epidemic (e.g., mask bearing, closure of schools, offices, businesses, places of worship, and entertainment, and prohibition of international travel and transportation) to assess its impact on the epidemic [50]. A high PHSM index, which combines several restrictive or even coercive measures, leads to a general disorganization of the society that generates stress with numerous psychological, social, and economic consequences. It prevents access to health facilities, many of which were temporarily closed due to containment and transport restriction measures. In addition, the lack of supplies, including sanitary equipment and medicines, and the failure of many businesses and trades, especially in the informal sector which is a crucial part of the economy in SSA, has led to concerns, logistical constraints, and a loosening of social links [27, 32, 55, 74, 84, 101, 108, 114].

Government restrictions have been responsible for the loss of huge numbers of jobs, especially in the informal sector, and resources that have led to a dramatic decline in purchasing power and prevented patients from accessing basic healthcare, which is their sole responsibility in most SSA countries [38, 57, 61]. Increased costs, due to logistical constraints and shortages, have only exacerbated patient insolvency [22, 33, 44, 62, 70, 94, 99, ].

Finally, rumors (infodemia) from all sources, and misconceptions or misinformation about diseases and healthcare, contributed to discouraging people from attending health centers, but also to school dropout and a range of psychosocial consequences such as mental health disorders, domestic violence, and prostitution [25, 34, 38, 56-58, 66, 72, 73, 95, 104, 105, 120].

### Structural factors

The overall unpreparedness of the health system (lack of availability of services, inadequate resources and equipment, insufficient testing and appropriate responses to the pandemic, including a shortage of personal protective equipment) was obvious and greatly accentuated the disorganization of the health system caused by conjunctural factors [14, 24-26, 37, 39, 44, 48, 50, 61, 63, 68, 69, 71, 74, 76, 78, 83, 84, 96, 99, 106, 108, 116, 118]. Added to this were the detour of resources [26, 28, 48, 119], an increase in overwork - due to the management of COVID-19 and the absenteeism of health workers who were ill, without transport or exhausted -, and the lack of training to face the new situations [51, 105].

The health information system and mandatory reporting were severely disrupted, preventing surveillance and the implementation of targeted interventions [118]. This may have led to an underestimation of the impact of COVID-19 on the overall health system and healthcare access. This situation has largely contributed to the demotivation of health personnel, their lack of attention to the public, and to increasing anxiety, and psychological disorders [51-53, 61, 118].

The delay or reduction of many healthcares, interruption of services, mitigation of transmission risk strategies or recommendations, difficulties in the supply of drugs and health products, high cost of the latter, and the shortage of essential medicines discouraged people who suspended their seek for healthcare [19, 24, 27, 57, 69, 78, 84, 94].

### Individual factors

Many factors have been put forward to explain the population's reluctance to travel, including visiting a health center. In addition to personal reasons, primarily the fear of contamination and resulting stigmatization, as well as the drop in income that limits many activities, conjunctural factors (lack of transportation, containment, curfews) and structural factors (insufficient human and material resources) explain the refusal to consult and the lack of access to healthcare. Fear of contamination, for themselves or their relatives, also concerned most health workers, especially those who had never experienced a major epidemic [37, 83, 118]. They often saw themselves as victims of stigma and discrimination [83, 97].

### Recommendations

Several studies have successfully tested strategies to address the observed deficiencies. Improved service delivery relies on technological innovations and adaptations, such as the use of self-tests, telemedicine through telephone or videoconferencing for diagnosis or prescription of drugs, and the use of drones for the distribution of health products [12, 19, 21, 26, 28, 29 37, 71]. In addition, reorganizing community services, arranging drug supplies, providing early non-pharmacological interventions (e.g., vector control), raising public awareness, and adapting treatment protocols were proving successful [15, 23, 26, 30, 47, 63, 70, 78, 84, 97, 110, 111, 113, 112].

Patient flows and supply of health products, including personal protective equipment, need to be regulated [12, 26, 46, 84]. In the event of service failures during the epidemic period, catch-up measures, including routine vaccinations, should be adopted [11, 58]. Collaboration between healthcare providers and public health services is a key element of the healthcare delivery system [74]. This includes the appropriate use of standardized health indicators and more accurate consideration of factors limiting the management of co-morbidities [20, 49].

Training of health workers in patient intake and management, and improvement of alternative diagnostic and therapeutic techniques, such as appropriate drug dispensing (e.g., provision of ART or TB treatment for several months) would help to anticipate difficulties that are bound to arise in accessing healthcare, in the supply of drugs, or health facility access [15, 23, 78, 113, 119].

The role of community health workers in the management of patients with conditions other than COVID-19, particularly in the most vulnerable slums and rural areas, requires stronger support and recognition [30, 85, 119]. Strengthening and expanding existing social protection schemes would reduce the deleterious consequences of epidemics in general and ensure more effective resilience, especially among vulnerable populations [47].

Misinformation and rumors should be controlled by appropriate information strategies with clear and simple messages, targeting community and opinion leaders, including religious ones, artists, and trusted individuals [20, 34, 38, 63].

Finally, legislation on domestic violence and the maintenance of constant surveillance, including in times of restricted movement and social disorganization, should be adjusted [75, 76].

This study was not intended to be exhaustive or to detail changes in healthcare access, but to identify the main impacts of COVID-19 on the organization and use of the health system in SSA. The outcomes of a literature review, such as this one, depend largely on the quality of the studies, i.e., their representativeness, the validity of the samples selected, and the relevance of the analyses performed. The procedures used to select the samples, the decision criteria, the presentation of the results and their discussion were not of the same quality according to the study, even if all the articles came from indexed peer-reviewed journals. In addition, health centers were not randomly selected, and some sample sizes were small. However, despite these limitations, the convergence of the raw data supported the validity of the results. The decline in healthcare access appeared in all studies with similar levels and kinetics over time, giving them credibility.

## Conclusion

The causes of the impact of COVID-19 on access to healthcare are multifactorial. However, restrictive government measures limiting travel, transport, supply, and the opening of public places, including shops and places of entertainment or worship, have blocked all social and economic activities with considerable deleterious consequences. The disorganization affected the whole of society, including the health system which was not prepared for it. In addition, at the individual level, the fear of contamination, reduction in income, economic and social disorder, in particular the loss of confidence in the health system, and stress increased by the infodemia explained the population's reluctance to healthcare access [34, 120]. The drastic measures taken at the beginning of the pandemic probably slowed and delayed its spread but at the cost of dramatic psychosocial and economic consequences.

Fortunately, although healthcare access was severely hampered (Table 2), the return to normal was rapid, showing the resilience of the health system, and a certain confidence of the population [40].

This epidemic, coming after many others, such as the one due to the Ebola virus [121], will be followed by new ones, which will require adapted responses that consider conjunctural and structural components such as the training of health personnel, appropriate equipment, available health products, precise procedures and information for the population. The general unpreparedness and lack of anticipation of the risk, forcing a disproportionate response, represents a socio-economic cost that will be less and less accepted by the populations in the future.
